# How to combine CTA, ^99m^Tc-WBC SPECT/CT, and [^18^F]FDG PET/CT in patients with suspected abdominal vascular endograft infections?

**DOI:** 10.1007/s00259-023-06309-x

**Published:** 2023-06-27

**Authors:** Chiara Lauri, Giuseppe Campagna, Francesco Aloisi, Alessandro Posa, Roberto Iezzi, Pasqualino Sirignano, Maurizio Taurino, Alberto Signore

**Affiliations:** 1 Nuclear Medicine Unit, Department of Medical-Surgical Sciences and of Translational Medicine, Sant’Andrea Hospital, “Sapienza” University of Rome, 00161 Rome, Italy; 2 Vascular Surgery Unit, Department of Clinical and Molecular Medicine, Sant’Andrea Hospital, “Sapienza” University of Rome, 00161 Rome, Italy; 3 Dipartimento Di Diagnostica Per Immagini, Radioterapia Oncologica ed Ematologia – Fondazione Policlinico Universitario A Gemelli IRCCS, Rome, Italy; 4 Diagnostic and Interventional Radiology Unit, Gemelli Molise Hospital, Campobasso, Italy; 5 Università Cattolica del Sacro Cuore, Rome, Italy; 6 Vascular Surgery Unit, Sant’Andrea Hospital, Department of General and Specialistic Surgery, Sapienza” University of Rome, 00161 Rome, Italy

**Keywords:** Vascular endograft infection, WBC scintigraphy, [^18^F]FDG PET/CT, CT angiography

## Abstract

**Purpose:**

We aimed at comparing ^99m^Tc-HMPAO white blood cells (^99m^Tc-WBC) scintigraphy, 18fluorine-fluorodeoxyglucose ([^18^F]FDG) positron emission tomography/computed tomography (PET/CT) and CT angiography (CTA) in patients with suspected abdominal vascular graft or endograft infection (VGEI). Moreover, we attempted to define a new visual score for interpreting [^18^F]FDG PET/CT scans aiming at increasing its specificity.

**Methods:**

We prospectively compared ^99m^Tc-WBC SPECT/CT, [^18^F]FDG PET/CT, and CTA in 26 patients with suspected abdominal VGEI. WBC scans were performed and interpreted according to EANM recommendations. [^18^F]FDG PET/CT studies were assessed with both qualitative (Sah’s scale and new visual score) and semi-quantitative analyses. CTA images were interpreted according to MAGIC criteria. Microbiology, histopathology or a clinical follow-up of at least 24 months were used to achieve final diagnosis.

**Results:**

Eleven out of 26 patients were infected. [^18^F]FDG PET/CT showed 100% sensitivity and NPV, with both scoring systems, thus representing an efficient tool to rule out the infection. The use of a more detailed scoring system provided statistically higher specificity compared to the previous Sah’s scale (*p* = 0.049). ^99m^Tc-WBC SPECT/CT provided statistically higher specificity and PPV than [^18^F]FDG PET/CT, regardless the interpretation criteria used and it can be, therefore, used in early post-surgical phases or to confirm or rule out a PET/CT finding.

**Conclusions:**

After CTA, patients with suspected late VGEI should perform a [^18^F]FDG PET/CT given its high sensitivity and NPV. However, given its lower specificity, positive results should be confirmed with ^99m^Tc-WBC scintigraphy. The use of a more detailed scoring system reduces the number of ^99m^Tc-WBC scans needed after [^18^F]FDG PET/CT. Nevertheless, in suspected infections within 4 months from surgery, ^99m^Tc-WBC SPECT/CT should be performed as second exam, due to its high accuracy in differentiating sterile inflammation from infection.

## Introduction

Endovascular aneurysm repair (EVAR) is, nowadays, a common procedure for the treatment of both abdominal aortic aneurysms (AAA) and thoracic aortic aneurysms (TAA), being less invasive and associated with lower morbidity and mortality compared to open repair [1, 2].

Although the incidence of vascular graft/endograft infection (VGEI) in the post-surgical period is low, ranging from 1 and 6% [3–7], it represents a fearsome and life-threatening complication. According to time elapsed from surgery, “early” VGEIs generally arise within the first 4 months and result from a possible contamination of the operating field;”late infections” usually occur 4 months after surgery and are mainly caused by hematogenous spread from a bacteremia, bacterial translocation, or iatrogenic contamination during catheterization [8–10].

Several factors may predispose the onset of an infection, including patient’s age, co-morbidities, and surgical-related risks (i.e., femoral/groin incision, the use of synthetic material, prolonged surgical time) [11].

Although broad range antibiotics represent a milestone for the treatment of an infection, conservative therapy may not be sufficient to completely eradicate the infection and the mortality rate within 2 years may approach 100% if an infected graft is left in situ [12]. Complete removal of the graft, debridement, and reconstruction, indeed, represent the best and definitive approach, although it is burdened by 18–30% mortality rate within one month from the re-intervention [13].

An accurate identification of infection, the definition of its extent, and the isolation of causative pathogen/s are, therefore, crucial for therapy decision making.

As recently suggested by the Management of Aortic Graft Infection Collaboration (MAGIC) [12], the diagnosis derives from the combination of clinical, biochemical, microbiological findings and imaging [7, 12, 14].

The clinical practice guidelines published in 2020 by the European Society of Vascular Surgery (ESVS) summarize the most effective strategies for both prevent and treat the infection [15]. More recently, European Association of Nuclear Medicine (EANM) published evidence-based guidelines specifically focused on radiological and nuclear medicine (NM) imaging modalities [16]. From these documents, computed tomography angiography (CTA) emerges as the first line modality when a VGEI is suspected.

Nevertheless, given its limited accuracy in early post-surgical phases and in low-grade processes [14, 16], NM modalities are strongly recommended to confirm or reject the diagnosis. Both radiolabelled white blood cells (WBC) scintigraphy and 18fluorine-fluorodeoxyglucose ([^18^F]FDG) positron emission tomography/computed tomography (PET/CT) are currently applied for imaging infections [17–19]. Despite several European guidelines have been published aiming to harmonize labelling procedures, acquisition protocols, and interpretation criteria for radiolabelled WBC scintigraphy [19–22], a similar standardization does not exist for [^18^F]FDG PET/CT imaging [23–25].

In particular, the limit between physiologic and pathologic [^18^F]FDG uptake in vascular grafts may be sometimes extremely difficult to assess and, despite different interpretation criteria have been recently proposed, a unanimous consensus has not been reached yet.

Similar to the study published by Keidar et al. in 2014 [26] in a large population of non-infected patients, we recently retrospectively assessed [^18^F]FDG biodistribution in oncologic patients with non-infected endovascular grafts, studied at different time-points after EVAR. We concluded that faint and diffuse [^18^F]FDG uptake can rule out the infection even in early post-surgical phases and, therefore, in patients showing low pre-test probability of infection, [^18^F]FDG PET/CT can be performed also immediately after surgery to exclude a VGEI [27].

The first aim of the present study was to prospectively compare the diagnostic performance of ^99m^Tc-WBC SPECT/CT, [^18^F]FDG PET/CT, and CTA in patients with suspected VGEI of abdominal tract. Second aim was to define a new visual scale for [^18^F]FDG PET/CT interpretation and to compare it with one of the most recently published by Sah et al. [28].

## Materials and methods

### Patient population

Patients with suspected abdominal VGEI, after the execution of a CTA scan, were prospectively studied with both [^18^F]FDG PET/CT and ^99m^Tc-HMPAO WBC scintigraphy + SPECT/CT in the NM Department of our institution, from January 2016 to December 2020. Depending on our waiting list, some patients performed ^99m^Tc-WBC before [^18^F]FDG PET/CT and some others started with [^18^F]FDG PET/CT.

Inclusion criteria were adults over 18 years; previous EVAR procedure for the exclusion of AAA with Endurant® grafts (Medtronic®, Santa Rosa, California); suspected VGEI according to clinical and biochemical findings described in MAGIC criteria (at least two minor criteria before imaging) [12]; information on final diagnosis; [^18^F]FDG PET/CT and ^99m^Tc-WBC SPECT/CT performed within 1 week and no more than 10 days after the execution of CTA; a follow-up of at least 24 months after NM examinations.

Exclusion criteria were patients with different kind of vascular grafts; patients with peripheral grafts; patients who did not perform both NM imaging modalities; lack of information on final diagnosis; inadequate follow-up.

We collected demographic information, time from surgery, laboratory tests, microbiology or histopathology, comorbidities, risk factors for general population (dyslipidemia, hypertension, cardio-vascular diseases, smoke habit and diabetes), and ongoing therapies.

NM scans and CTA were assessed by three and two readers with experience in VGEIs imaging, respectively. All the readers were blinded to clinical information except for the time from surgery.

Since the study comprises the analysis of routinely performed imaging modalities, the local ethic committees waived the need for approval.

### ^99m^Tc-WBC SPECT/CT

Autologous leukocytes were labelled with ^99m^Tc-HMPAO according to EANM guidelines [21, 22] by using Leukokit® (GiPharma, Italy). Labelling efficiency was calculated in order to verify the quality of the product (normal range: 40–80%). The mean administered dose of ^99m^Tc-WBC was approximately 555–740 MBq (15–20 mCi).

A dynamic acquisition (40 frames: 4 s/frame) was performed in anterior–posterior view immediately after the intravenous injection (i.v.) of labelled leukocytes for the first 5 min to visualize the vasculature. Static planar anterior–posterior and oblique images (using 128 × 128 matrix) were acquired using dual-head SPECT gamma camera (Forte, Philips). Planar images of the abdomen were acquired at three times point (“early,” “delayed,” and “late” images), with times corrected for isotope decay, respectively at 30 min (100 s), 2 h (133 s), and 20 h (1007 s) post injection (p.i.). All images were displayed in absolute counts using the same intensity colour scale [22].

SPECT/CT was performed after 20 h p.i. to correctly localize the uptake and to accurately evaluate its extent. SPECT of abdomen was acquired using a 128 × 128 matrix, 360° rotation, 6° steps, and an acquisition of 30 s/frame. A low-dose CT transmission scan of the same field of view was acquired with the following parameters: 140 kV, 90 mA, 0.8/s tube rotation, and 5 mm thickness. SPECT and CT images were fused using Hermes Hybrid Viewer™ workstation (Hermes Medical Solutions), and the scans were analysed in the three axes.

The scans were classified as:

- negative for infection if no abnormal uptake was detected or if the intensity and/or extent of the uptake in late images was the same or decreased over time compared to delayed images;
- positive for infection if the uptake in delayed images showed an increase over time, in terms of intensity and/or extension, in late images [21, 22].

### [^18^F]FDG PET/CT

PET images were acquired, for 2.5 min per bed position from head to mid-thigh, 50–60′ p.i. of 2.5–5 MBq/kg of [^18^F]FDG with a dedicated hybrid PET/CT tomography (Gemini, Philips) combining a third-generation multi-slice spiral CT scanner with a full-ring PET scanner (bismuth germinate crystals) until July 2018 and with hybrid PET/CT Biograph (Siemens, Germany) with multi-slice spiral CT scanner with a 5-ring PET scanner (lutetium oxyorthosilicate crystals) from September 2018. Low-dose CT scan for attenuation correction and anatomic location was performed with the following parameters: 140 kV, 90 mA, 0.8/s tube rotation, and 5 mm thickness. Corrected PET images were automatically fused with CT images and displayed in maximum intensity projections (MIP) in axial, coronal, and sagittal planes.

Qualitative assessment was performed by using the 5-point visual grading score proposed by Sah et al. [28]:

- Score 1: normal background activity;
- Score 2: mild and diffuse [^18^F]FDG uptake along the graft (less than twice the blood pool activity in the ascending aorta);
- Score 3: focal and mild, or strong and diffuse [^18^F]FDG uptake along the graft (more than twice the blood pool activity in the ascending aorta);
- Score 4: focal and intense [^18^F]FDG uptake (± diffuse);
- Score 5: focal and intense [^18^F]FDG uptake plus fluid collections/abscess formation.

According to this classification, the scan was interpreted as positive from Score 3.

**Fig. 1:**
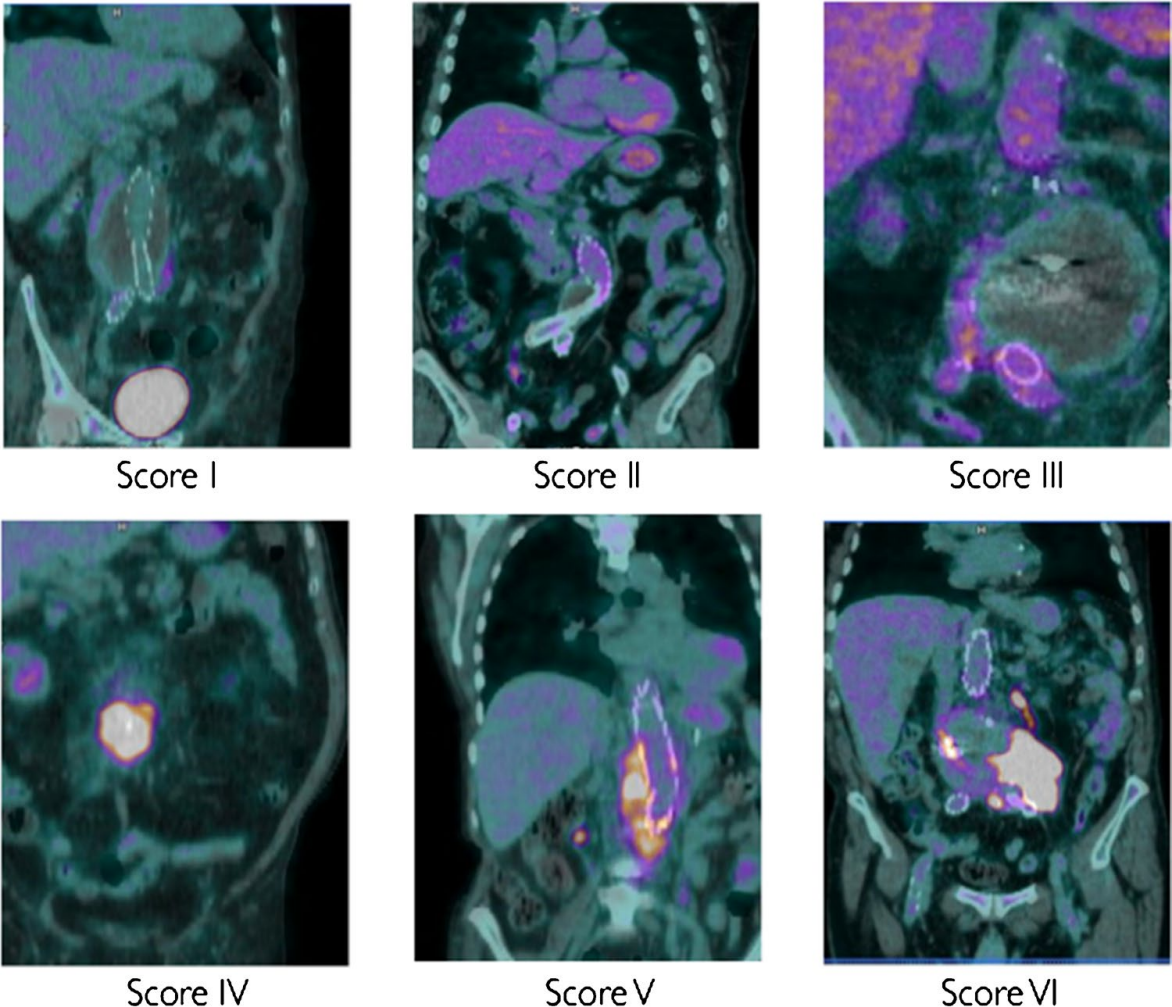
Examples of new visual grading scale

Moreover, we also attempted to define a novel visual scoring system that takes into consideration the pattern distribution of the uptake, rather than its intensity (Fig. 1):

- Score I: normal background activity;
- Score II: homogeneous and diffuse [^18^F]FDG uptake of any intensity along the graft (diffuse uptake which is uniformly distributed along the graft);
- Score III: non-homogeneous and diffuse [^18^F]FDG uptake of any intensity along the graft (diffuse uptake which is not uniformly distributed along the graft, presenting areas with higher uptake and areas with lower uptake but without clear focal uptake detectable in the context);
- Score IV: focal [^18^F]FDG uptake of any intensity (one or more clearly visible foci of any intensity on the graft without any other diffuse uptake);
- Score V: focal + diffuse [^18^F]FDG uptake of any intensity (presence of both homogeneous and non-homogeneous distribution with ≥ 1 focal areas clearly detectable in the context);
- Score VI: uptake extended to peri-prosthetic tissues (presence of psoas abscesses, vertebral disk involvement, loco-regional lymph-nodes, enteric fistulae…).

According to this new classification, the scan was interpreted as positive from Score IV.

Both scoring systems were used for interpreting all the [^18^F]FDG PET/CT scans and were compared according to final diagnosis.

For semi-quantitative assessment, we collected:

- maximum and mean standardized uptake value (SUVmax and SUVmean), drawing a 50-pixel circular region of interest (ROI) over the site with maximum [^18^F]FDG uptake covering the whole vessel;
- target/background (T/B) ratio, drawing a 20-pixel circular ROI in the inner of first tract of descending aorta (approximately in the tract between V and VI dorsal vertebra), as reference for blood pool background. The following formula: SUVmean target/SUVmean background was used for T/B ratio calculation.

### CTA

CTA examinations were mainly performed in Radiology Department of our Institution and were analysed in blind by 2 radiologists with experience in VGEIs.

CTA examinations were performed using a 64-slice multidetector-row CT (Lightspeed VCT XT, GE), with the following parameters: 120 kV, 130 mAs, 15 mm/rotation table speed, and a 0.5-s gantry rotation time. A 2.5-mm slice width and a 2.5-mm reconstruction interval were used for unenhanced acquisitions; A 0.6-mm slice width and a 0.6-mm reconstruction interval were used for contrast-enhanced (CE) scans (arterial and venous/delayed phases) acquisitions.

All CE-CT scans were performed by injecting 80 mL of iodinate non-ionic contrast medium (Iomeprol 400 mgI/mL, Bracco, Italy), followed by a 40-mL of saline. Dynamic bolus tracking was used to obtain the correct timing of arterial acquisition, positioning a small circular ROIs in the aortic arch — far away from parietal calcifications and from any metallic graft which could hinder the correct Hounsfield unit (HU) measurements, setting the threshold to 150 HU and minimum scan delay (usually 5–6 s) with an inter-scan delay of 1 s; a 60-s delayed/venous phase was acquired after the arterial phase.

Images were interpreted according to MAGIC criteria [12]. According to this classification, major criteria were:

- increasing peri-graft gas volume on serial CT images.
- peri-graft gas at ≥ 7 weeks post-implantation.
- peri-graft fluid collection at ≥ 3 months post-implantation.

Any other radiological sign was considered as a minor criterion:

- suspicious peri-graft fluid or gas.
- soft tissue inflammation.
- aneurysm sac expansion.
- anastomotic leakage or pseudoaneurysm formation.
- focal bowel thickening or graft-enteric erosion/fistula.
- peri-graft discitis/osteomyelitis.

CTA examinations were classified as:

- negative: if no major or minor criteria were met.
- positive: if at least one major criterion was met.

Doubtful cases (i.e., when only minor criteria were present) were solved by a consensus.

Unenhanced and CE-CT images were evaluated by using dedicated workstations (Advantage Workstation 4.1, GE) to perform multiplanar reconstructions, MIPs, and volume rendering techniques, with the aim of better evaluate native vessels and vascular grafts.

### Final diagnosis and follow-up

According to imaging, patients were classified as “infected,” if at least two out of three modalities were concordantly positive and “non-infected” if at least two out of three modalities were concordantly negative.

Imaging results were, then, compared with gold standard for calculating the diagnostic performance of each modality. Microbiological confirmation (fluid aspirations, haemocultures, or intra-operative sampling), histopathological findings, in patients eligible to a surgical procedure, and a clinical follow-up of at least 24 months after NM examinations were used as gold standard for final diagnosis.

### Statistical analysis

Continuous variables are showed as mean ± standard deviation (SD) and 95% confidence interval (CI) when normally distributed, or as median (min to max) and 95%CI. Categorical variables are expressed as absolute frequencies and percentages — *n* (%).

The comparisons of the categorical variables between non-infected patients *vs.* infected patients were evaluated by *X*^2^ or Fisher exact tests (when expected frequencies were < 5); while for the continuous variables, we used *t* Student test (normality verified) or Mann–Whitney test (if normality failed). Normality of the distribution of continuous variables was verified by Shapiro–Wilk test and checking *Q*-*Q* plot.

Sensitivity, specificity, diagnostic accuracy, positive predictive value (PPV), and negative predictive value (NPV) of radiolabelled WBC scintigraphy, [^18^F]FDG PET/CT, and CTA were calculated by the SAS software and compared performing *z*-test for the equality of two proportions.

The association between [^18^F]FDG uptake and risk factors for general population (dyslipidemia, hypertension, cardio-vascular diseases, smoke habit, and diabetes) was evaluated by generalized linear mixed model (GLIMMIX) with Gaussian distribution.

The correlations between SUVmax, SUVmean, T/B ratios, and time elapsed from surgery were evaluated by Spearman’s coefficient *ρ* because variables were not normally distributed.

The cut-off values for SUVmax, SUVmean, and T/B ratios were obtained using three methods: (1) Youden index method (*J*); (2) minimum distance (0.1) namely point closest-to-(0.1) corner in the ROC curve; (3) minimum difference in modulus of sensitivity and specificity.

Cut-off values were chosen when at least 2 out of 3 methods provided the same result.

A *p* < 0.05 was considered statistically detectable. Statistical analysis was performed using SAS version 9.4 and JMP PRO version 17 (SAS Institute, Cary, NC, USA).

## Results

Twenty-six patients (21 males, 5 females; mean age 73.69 ± 7.14 years) with suspected abdominal VGEI have been studied with both [^18^F]FDG PET/CT and ^99m^Tc-WBC SPECT/CT, within 10 days after the execution of a CTA. CTA images were not available in 4 patients who performed the exam in a different centre. Reports of these CTA scans were negative for infection in 3 cases and positive in one case. Nevertheless, these 4 patients were considered only for PET/CT and SPECT/CT.

Prior to imaging, 23 patients showed two minor MAGIC criteria (fever and raised CRP/ESR), and two of them became positive at blood culture/fluid drainage only after the execution of CTA, WBC scan, and [^18^F]FDG PET/CT; 3 patients showed three minor criteria (fever, raised CRP/ESR, and positive blood culture) before imaging.

According to time from surgery (less than 4 months), an “early infection” was suspected in 5 patients and a “late infection” in the other 21. Seven patients were under antibiotic therapy at time of studies (started from 2 to 5 days before imaging).

An infection was diagnosed in 11 out of 26 patients (42.31%). Final diagnosis was achieved by the isolation of causative pathogen in 8 patients — blood culture (n.4), graft removal (n.3), and fluid drainage (n.1) — and by the evidence of leukocyte infiltration or an abscess, at histology performed during surgery, in the other 3 patients. A clinical follow-up of at least 24 months was used to confirm the absence of infection in patients with at least two negative imaging modalities.

As shown in Table 1, no differences between patients with or without proven infection were found in terms of ESR, CRP, and WBC count.

**Table 1:** Comparison between non-infected patients and infected patients

Parameter	Non-infected patients Mean ± SD (95%CI)	Infected patients Mean ± SD (95%CI)	*p*
Sex* (male/female)	13 (61.90)/2 (40.00)	8 (38.10)/3 (60.00)	0.62
Age (years)	73.07 ± 6.41 (69.52 to 76.61)	74.54 ± 8.27 (68.99 to 80.10)	0.61
ESR (mm/h)	33.67 ± 22.99 (19.06 to 48.27)	56.50 ± 23.33 (32.02 to 80.98)	0.07
WBC count (10 × 3/μL)	9.38 ± 3.79 (6.97 to 11.79)	12.85 ± 4.22 (8.95 to 16.75)	0.08
CRP (mg/dL)	10.92 ± 8.53 (5.19 to 16.65)	14.25 ± 7.78 (7.74 to 20.75)	0.40
Time elapsed from surgery (months)**	24 (1 to 144) (4 to 72)	36 (9 to 132) (16 to 96)	0.39
SUVmax**	4.35 (2.19 to 12.01) (3.26 to 8.50)	5.96 (3.80 to 22.50) (4.66 to 11.39)	0.07
SUVmean**	2.50 (1.74 to 6.15) (1.93 to 3.06)	3.55 (2.82 to 8.93) (2.84 to 4.80)	**0.01**
T/B ratio**	1.44 (0.77 to 5.52) (1.08 to 2.13)	2.07 (1.44 to 6.86) (1.94 to 5.62)	**0.02**
Smoke* (yes/no)	5 (71.43)/10 (52.63)	2 (28.57)/9 (47.37)	0.66
Hypertension* (yes/no)	10 (62.50)/5 (50.00)	6 (37.50)/5 (50.00)	0.69
Dyslipidemia* (yes/no)	5 (62.50)/10 (55.56)	3 (37.50)/8 (47.06)	0.99
Diabetes* (yes/no)	2 (50.00)/13 (59.09)	2 (50.00)/9 (40.91)	0.99
CV diseases* (yes/no)	9 (69.23)/6 (46.15)	4 (30.77)/7 (53.85)	0.23

*ESR* erythrocyte sedimentation rate, *WBC* white blood cells, *CRP* C-reactive protein, *CV* cardiovascular, *SUVmax* maximum standardized uptake value, *SUVmean* mean standardized uptake value, *T/B* target/background ratio

*Data presented as absolute frequency (%); **data presented as median value (min–max) and 95% CI

values in bold are statistically significant

Two patients with concordantly positive CTA and [^18^F]FDG PET/CT and high clinical suspicion underwent to surgery, but no microbes were detected after microbiological culture of histological samples (patient n. 17 and 19; Table 2).

**Table 2:** Results of CTA, radiolabelled WBC scintigraphy, and [^18^F]FDG PET/CT according to final diagnosis

ID	Months from surgery	MAGIC criteria (before imaging)	CTA	^99m^Tc-WBC scan	[^18^F]FDG PET/CT					Diagnosis (microbiology)		Therapy
					Sah’s score	New score	SUVmax	SUVmean	T/B ratio			
1	9	2 minor	TP	TP	TP (5)	TP (6)	5.22	2.84	2.07		VGEI (Stenotrophomonas maltophilia from haemoculture)	Antibiotic
2	10	3 minor	FN	TP	TP (4)	TP (6)	4.52	2.82	3.07		VGEI (Histology positive*)	Surgery
3	18	2 minor	TP	FN	TP (5)	TP (6)	3.8	3.02	1.86		VGEI (Candida Tropicalis from drainage)	Antibiotic
4	20	3 minor	FN	TP	TP (5)	TP (6)	4.66	3.2	2.67		VGEI (Candida Albicans from haemoculture)	Antibiotic
5	36	2 minor	TP	TP	TP (5)	TP (6)	22.5	8.93	5.62		VGEI (Staphilococcus Epidermidis)	Surgery
6	39	3 minor	FN	TP	TP (5)	TP (6)	5.96	2.83	1.44		VGEI (Bacteroides Fragilis from haemoculture)	Antibiotic
7	48	2 minor	n.a	TP	TP (5)	TP (6)	5.75	3.86	4.83		VGEI (Histology positive*)	Surgery
8	132	2 minor	TP	TP	TP (3)	TP (4)	9.61	4.8	6.86		VGEI (Histology positive*)	Surgery
9	84	2 minor	TP	TP	TP (5)	TP (6)	6.97	4.54	2.07		VGEI (Staphilococcus Epidermidis)	Surgery
10	96	2 minor	TP	TP	TP (5)	TP (6)	11.39	3.55	1.94		VGEI (Enterococcus faecium from haemoculture)	Antibiotic
11	16	2 minor	TP	FN	TP (5)	TP (6)	9.76	3.66	1.94		VGEI (Pseudomonas Aeruginosa)	Surgery
12	4	2 minor	n.a	TN	TN (2)	TN (1)	3.31	2.5	1.44		No infection detected	FU without therapy
13	60	2 minor	TN	TN	FP (3)	TN (2)	4.35	1.93	1.05		No infection detected	FU without therapy
14	2	2 minor	TN	TN	FP (4)	FP (5)	4.29	2.68	1.81		No infection detected	FU without therapy
15	2	2 minor	FP	TN	TN (2)	TN (2)	2.19	1.79	1.81		No infection detected	FU without therapy
16	72	2 minor	TN	TN	TN (2)	TN (2)	3.26	2.12	1.18		No infection detected	FU without therapy
17	7	2 minor	FP	TN	FP (4)	FP (5)	7.5	5.69	5.52		No infection detected	Surgery
18	24	2 minor	n.a	TN	FP (3)	TN (2)	4.56	2.7	0.77		No infection detected	FU without therapy
19	24	2 minor	FP	TN	FP (5)	FP (6)	8.7	6.15	5.04		No infection detected	Surgery
20	132	2 minor	TN	TN	TN (2)	TN (2)	2.4	1.89	1.04		No infection detected	FU without therapy
21	4	2 minor	TN	TN	FP (3)	TN (3)	8.5	2.45	2.13		No infection detected	FU without therapy
22	1	2 minor	TN	TN	TN (2)	TN (2)	3.71	1.98	1.32		No infection detected	FU without therapy
23	53	2 minor	TN	TN	TN (2)	TN (2)	2.75	1.74	1.26		No infection detected	FU without therapy
24	13	2 minor	n.a	TN	FP (5)	FP (6)	9.65	5.9	2.62		No infection detected	FU without therapy
25	79	2 minor	TN	TN	FP (3)	TN (3)	12.01	3.06	2.11		No infection detected	FU without therapy
26	144	2 minor	TN	TN	FP (3)	TN (3)	5.34	2.52	1.08		No infection detected	FU without therapy

Minor MAGIC criteria were fever and high CRP and/or ESR and/or positive blood culture

*n.a.* not available, *TP* true positive, *TN* true negative, *FP* false positive, *FN* false negative, *FU* follow-up

*Histology positive for leukocytes/abscess but no bacteria were isolated

^99m^Tc-WBC scintigraphy provided 9 true positive (TP) results, 15 true negative (TN) results, and no false positive (FP) findings, even in patients studied at early phases after surgery.

Patient numbers 18 and 24, in which we had discordant WBC scan and [^18^F]FDG PET/CT, after consultation with vascular surgeons, and also taking into consideration the negative CTA report, were strictly followed-up. In particular, the high activity observed in patient n. 24 at [^18^F]FDG PET/CT was attributed to macrophages activation in the aneurysmatic sac and surrounding tissues due to pre-operatory bleeding.

**Fig. 2:**
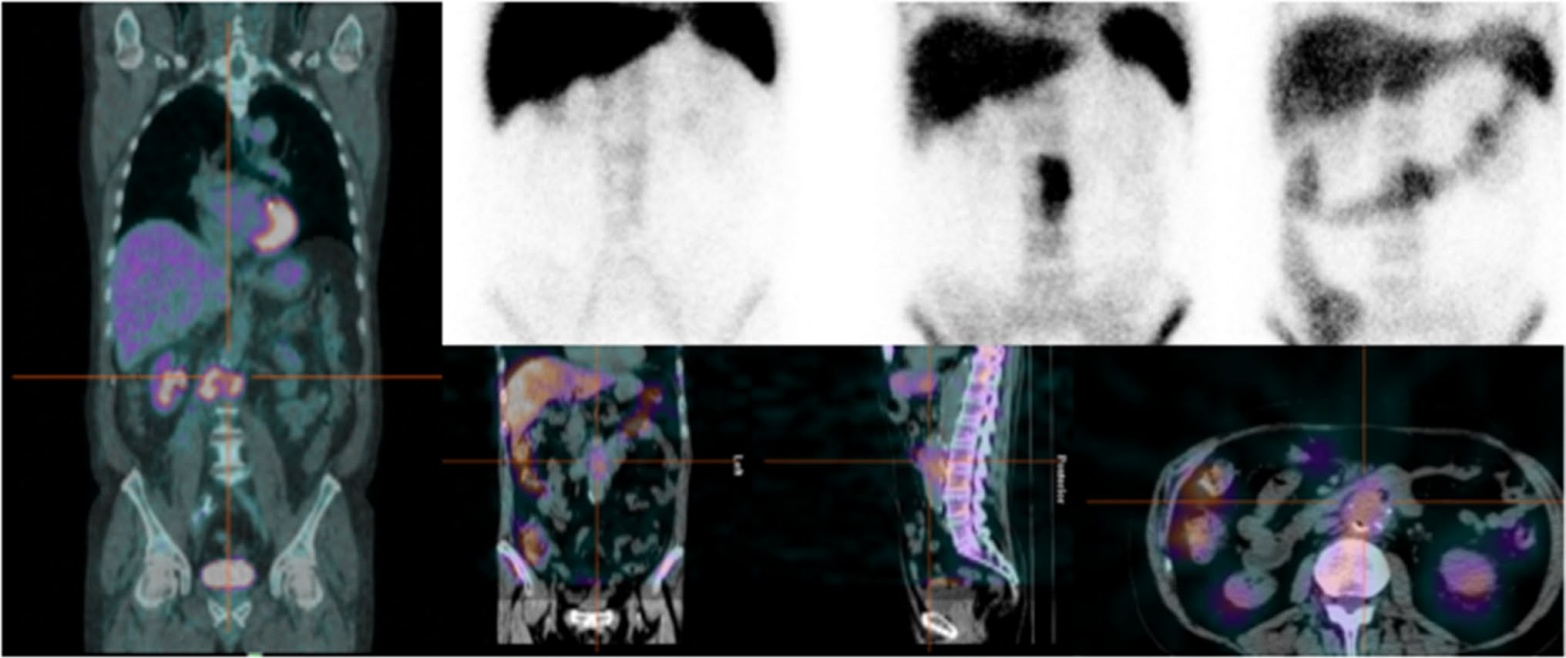
Example of concordantly positive PET/CT (left panel) and ^99m^Tc-WBC scintigraphy (planar images in upper right panel, 30’, 2 h and 20 h after injection; SPECT/CT images (in lower panel) in a patient with proved infection by Enterococcus faecium

**Fig. 3:**
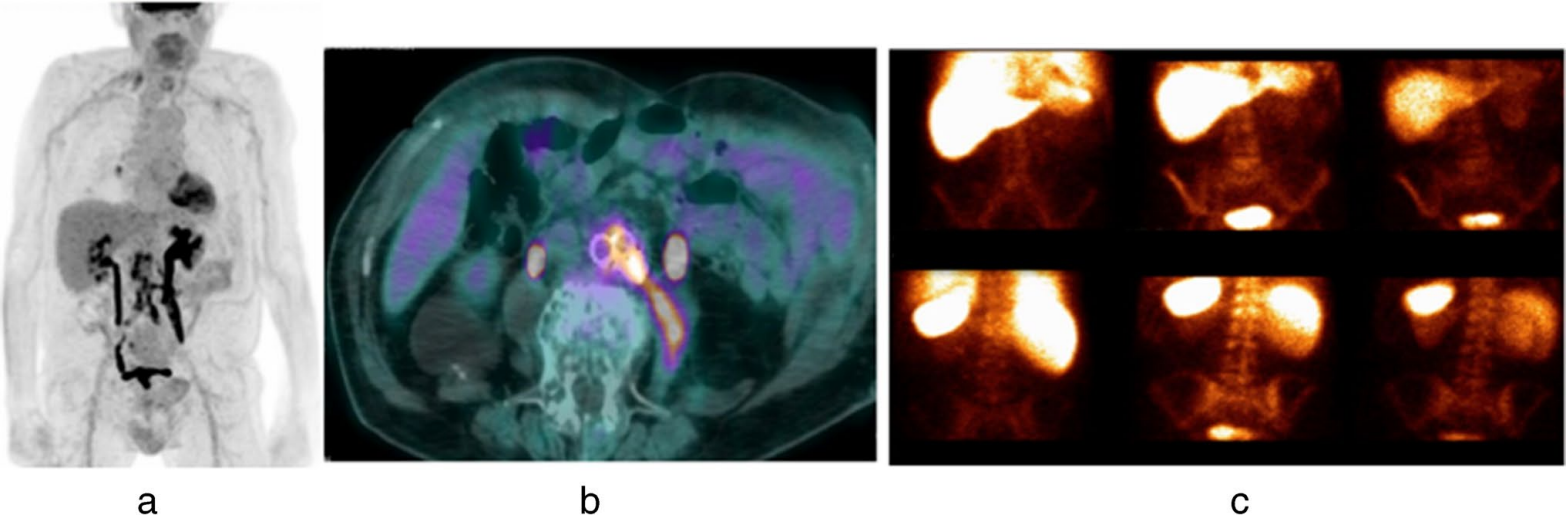
Example of TP at PET/CT and FN at ^99m^Tc-WBC scan. MIP images (**a**) and axial views of fused PET/CT images (**b**) were consistent with VGEI (score VI for the new scale and 5 for Sah scale) due to the presence of increased activity extended to peri-graft tissues (left psoas muscle). Planar antero-posterior views of ^99m^Tc-WBC scan (**c**) were negative. After NM examinations, *Candida tropicalis* was isolated in this patient

SPECT/CT allowed to accurately localize the infection into the graft and to evaluate its extent (Fig. 2). Two false negative (FN) results were observed in 2 patients with a proven infection by *Candida tropicalis* (patient n. 3; Fig. 3) and *Pseudomonas aeruginosa* (patient n. 11), respectively. No FN results were observed in the 7 patients under antibiotic therapy (three infected and four non-infected). This resulted in a sensitivity, specificity, accuracy, PPV, and NPV of 82%, 100%, 92%, 100%, and 88%, respectively (Table 3).

**Table 3:** Diagnostic performance of ^99m^Tc-WBC scintigraphy, [^18^F]FDG PET/CT and CTA

Clinimetric parameters	^99m^Tc-WBC scintigraphy	[^18^F]FDG (Sah’s score)	[^18^F]FDG (new score)	CTA
Sensitivity (95%CI)	81.8 (59.0 to 100)	100 (100 to 100)	100 (100 to 100)	70.0 (41.6 to 98.4)
Specificity (95%CI)	100 (100 to 100)	40.0 (15.2 to 64.8)	73.3 (50.9 to 95.7)	75.0 (50.5 to 99.5)
Accuracy (95%CI)	92.3 (82.1 to 100)	65.4 (47.1 to 83.7)	84.6 (70.6 to 98.5)	72.7 (54.1 to 91.3)
PPV (95%CI)	100 (100 to 100)	55.0 (33.2 to 76.8)	73.3 (50.9 to 95.7)	70.0 (41.6 to 98.4)
NPV (95% CI)	88.2 (72.9 to 100)	100 (100 to 100)	100 (100 to 100)	75.0 (50.5 to 99.5)

*PPV* positive predictive value, *NPV* negative predictive value

### Comparisons

^99m^Tc-WBC scintigraphy *vs.* Sah’s score, *p* < 0.0001 and ^99m^Tc-WBC scintigraphy *vs.* new score, *p* = 0.03 for both specificity and PPV; new score *vs.* Sah’s score *p* = 0.049 for specificity (z-test for proportions)

The use of Sah’s scale for [^18^F]FDG PET/CT interpretation [28], provided 11 TP, 6 TN, 9 FP, and no FN results, thus resulting in a sensitivity, specificity, accuracy, PPV, and NPV of 100%, 40%, 65.4%, 55%, and 100%, respectively (Table 3).

With the new visual scale, we obtained 11 TP, 11 TN, 4 FP, and no FN results, thus providing a sensitivity, specificity, accuracy, PPV, and NPV of 100%, 73%, 84.6%, 73.3%, and 100%, respectively (Table 3). No FN results were detected with both scores, even in patients with ongoing antibiotic treatment.

As shown in Table 2, the two scales were concordant in 21 cases. Five patients 5, considered positive (score 3) by Sah’s scale, were correctly classified as negative by using the new scoring system.

Infected patients showed higher SUVmean and T/B ratios compared to non-infected patients (*p* = 0.01 and *p* = 0.02, respectively), whereas no differences were found in terms of SUVmax (*p* = 0.07) (Table 1). However, the value of SUVmax or SUVmean did not help in the diagnostic process. The calculated cut-off value for SUVmax was 4.52 for diagnosing an infection with an overall sensitivity and specificity of 90.9% and 53.3%, respectively, and an AUC of 72.0 (95%CI: 51.0 to 92.0). The cut-off value for SUVmean was 2.82, with an overall sensitivity and specificity of 100% and 73.3%, respectively, and an AUC of 79.0 (95%CI: 60.0 to 99.0). The cut-off value for T/B ratio was of 1.86, with an overall sensitivity and specificity of 90.9% and 66.7%, respectively, and an AUC of 76.0 (95%CI: 57.0 to 95.0).

**Fig. 4:**
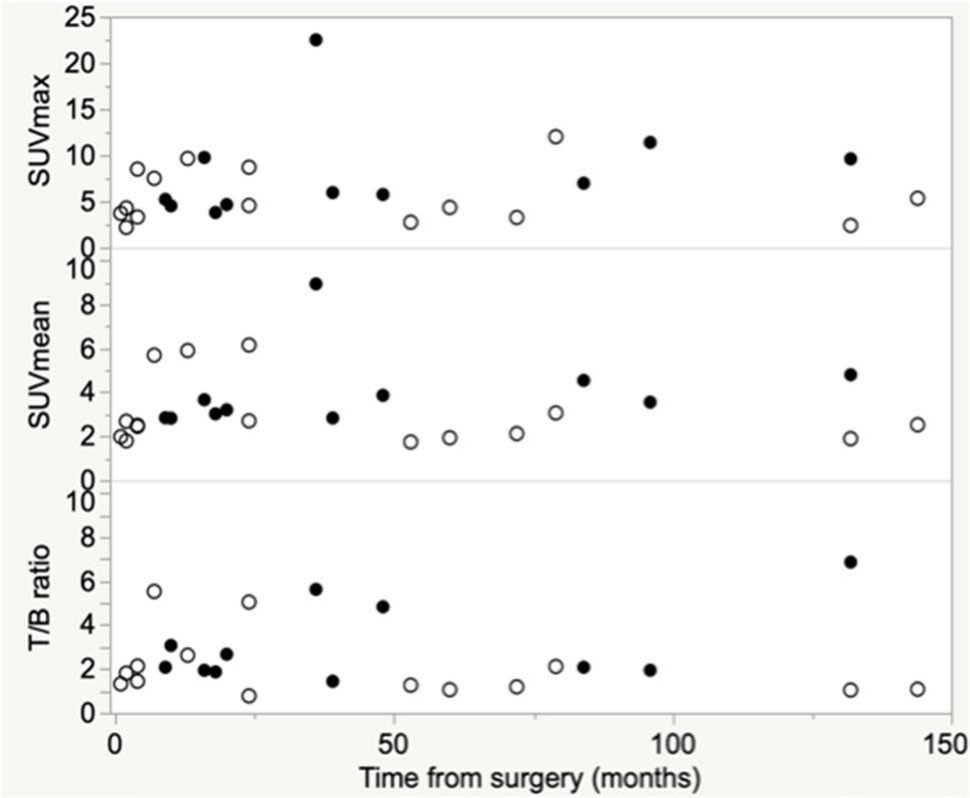
Relationship between SUVmax, SUVmean and T/B ratios and time from surgery in patients with suspected VGEI. White dots represent non-infected patients; black dots identify patients with proven infection. Neither SUVmax, nor SUVmean, nor T/B ratios were correlated with time from surgery

Moreover, SUVmax, SUVmean, or T/B ratios did not show any correlation with time elapsed from surgery (Fig. 4).

No association between [^18^F]FDG uptake and risk factors for general population reported in Table 1 was detected.

The interpretation of CTA images was discordant in only three cases that were considered “doubtful” based on minor criteria (suspicious peri-graft fluid and soft tissue inflammation). These equivocal cases were discussed in consensus by the two readers and finally classified as negative (all TN at gold standard). CTA was positive in 10 out of 22 patients for the presence of at least one major MAGIC criterion (3 patients with peri-graft gas 7 weeks post-implantation and 7 patients with peri-graft fluid collections 3 months after surgery). It was TP in 7 patients, FN in 3, FP in 3, and TN in 9 patients, thus resulting in a sensitivity, specificity, accuracy, PPV, and NPV of 70%, 75%, 72.7%, 70%, and 75%, respectively (Table 3).

### Clinical impact of multimodal imaging approach on the therapeutic management

According to imaging results, and before performing microbiology/histology, patients were considered infected when at least two modalities were positive. Following this criterion, 13 patients were managed as infected and treated with antibiotic therapy and/or with surgery, depending on their clinical conditions and surgical-related risk. A prolonged antibiotic treatment, based on the isolated microorganism, was started in 5 patients, whereas surgery was performed in 8 patients. Two out of these 8 patients (patients n. 17 and 19), with positive CTA and [^18^F]FDG PET/CT at both scoring systems did not show infection at microbiological examination of the prostheses after the explant. It is worthwhile to mention that these patients were both negative at ^99m^Tc-WBC scintigraphy.

Based on imaging results, 13 patients were considered non-infected and were strictly followed up by vascular surgeons with clinical examinations, biochemical assessment and further imaging studies. At the end of this study, all the population completed more than 24 months of follow-up (mean range 58.0 ± 35.5 months) and none of the non-infected patients showed signs or symptoms of VGEI.

The CTA alone would have determined a wrong management in 6 (3 FN and 3 FP) out of 22 patients (27.3%). Overall, the addition of NM imaging modality allowed changing the management in a non-negligible number of patients.

After the initial CTA scan, the order of NM examination was based on our waiting list and availability at the time of the study; therefore, some patients performed ^99m^Tc-WBC scintigraphy as second modality and [^18^F]FDG PET/CT as third examination, and some others followed the opposite approach.

Aiming at defining which combination would result in the best management in our population, we tested three different hypotheses (Tables 4, 5, 6, 7):

1. CTA + ^99m^Tc-WBC SPECT/CT: these two modalities were discordant in 8 out of 22 cases (36.4%). All the 3 FN and 3 FP results at CTA were correctly identified as infected and non-infected, respectively, by ^99m^Tc-WBC scintigraphy. On the other hand, ^99m^Tc-WBC scintigraphy missed the diagnosis of infection in 2 cases, which were correctly identified by CTA. In both cases, the addition of [^18^F]FDG PET/CT, as a third examination, allowed a correct re-classification of the patients, thus allowing to plan the correct treatment. However, this approach resulted in the overtreatment of two patients with a FP CTA and [^18^F]FDG PET/CT and a TN ^99m^Tc-WBC scintigraphy.

**Table 4:** First hypothesis

ID	Months from surgery	CTA	^99m^Tc-WBC scan	[^18^F]FDG PET/CT		Diagnosis	Therapy
				Sah’s scale	New scale		
2	10	FN	TP	TP	TP	VGEI	Surgery
3	18	TP	FN	TP	TP	VGEI	Antibiotic
4	20	FN	TP	TP	TP	VGEI	Antibiotic
6	39	FN	TP	TP	TP	VGEI	Antibiotic
11	16	TP	FN	TP	TP	VGEI	Surgery
15	2	FP	TN	TN	TN	No infection detected	FU without therapy
17	7	FP	TN	FP	FP	No infection detected	Surgery
19	24	FP	TN	FP	FP	No infection detected	Surgery

**Table 5:** Second hypothesis

ID	Months from surgery	CTA	^99m^Tc-WBC scan	[^18^F]FDG PET/CT		Diagnosis	Therapy
				Sah’s scale	New scale		
2	10	FN	TP	TP	TP	VGEI	Surgery
4	20	FN	TP	TP	TP	VGEI	Antibiotic
6	39	FN	TP	TP	TP	VGEI	Antibiotic
13	60	TN	TN	FP	TN	No infection detected	FU without therapy
14	2	TN	TN	FP	FP	No infection detected	FU without therapy
15	2	FP	TN	TN	TN	No infection detected	FU without therapy
21	4	TN	TN	FP	TN	No infection detected	FU without therapy
25	79	TN	TN	FP	TN	No infection detected	FU without therapy
26	144	TN	TN	FP	TN	No infection detected	FU without therapy

**Table 6:** Third hypothesis

ID	Months from surgery	CTA	^99m^Tc-WBC scan	[^18^F]FDG PET/CT		Diagnosis	Therapy
				Sah’s scale	New scale		
2	10	FN	TP	TP	TP	VGEI	Surgery
4	20	FN	TP	TP	TP	VGEI	Antibiotic
6	39	FN	TP	TP	TP	VGEI	Antibiotic
14	2	TN	TN	FP	FP	No infection detected	FU without therapy
15	2	FP	TN	TN	TN	No infection detected	FU without therapy

**Table 7:** Possible strategies of combination and their impact on patients’ management in our population

Hypothesis	Initial strategy (n. of exams performed)	Third imaging modality (n. of exams required for confirmation)	Patients’ management
1	CTA (22) + ^99m^Tc-WBC scan (22)	[^18^F]FDG PET/CT (5)	Overtreatment in 2 patients (FP at both CTA and [^18^F]FDG)
2	CTA (22) + [^18^F]FDG PET/CT with Sah scale (22)	^99m^Tc-WBC (8)	All the 8 patients were correctly diagnosed by WBC
3	CTA (22) + [^18^F]FDG PET/CT with the new scale (22)	^99m^Tc-WBC (4)	All the 4 patients were correctly diagnosed by WBC

2. CTA + [^18^F]FDG PET/CT (interpreted according to Sah’s scale): these two modalities were discordant in 9 out of 22 cases (41%). All the 3 FN results at CTA were correctly identified as infected by [^18^F]FDG PET/CT. [^18^F]FDG PET/CT was able to rule out the infection in one FP result at CTA but it was FP in 5 patients with a TN CTA. The addition of ^99m^Tc-WBC scintigraphy, as a third examination, allowed planning the most appropriate therapeutic strategy in all these five patients.

3. CTA + [^18^F]FDG PET/CT (interpreted according to the new scale): these two modalities were discordant in 5 out of 22 cases (23%). Again, [^18^F]FDG PET/CT was able to provide the correct diagnosis in 3 FN and in one FP at CTA but it was FP in one patient with a TN CTA scan. In this patient, ^99m^Tc-WBC scintigraphy allowed to definitively exclude an infection.

## Discussion

To the best of our knowledge, this is the first and the largest prospective study directly comparing three imaging modalities, with microbiology as gold standard, in patients with suspected abdominal VGEI according to MAGIC criteria. Although CTA and NM examinations take part of these criteria, in clinical practice, the appeal to imaging, with CTA as first line modality, is usually performed only when an infection is clinically suspected and after the execution of laboratory/microbiological tests. We, therefore, selected patients with at least 2 minor clinical and laboratory criteria before imaging, aiming at comparing CTA, WBS scan + SPECT/CT, and [^18^F]FDG PET/CT and at defining which combination of imaging modalities would result in a better management of the patients.

Three previously published studies comparing ^99m^Tc-WBC scintigraphy and [^18^F]FDG PET/CT achieved different results and conclusions, mainly depending on acquisition protocols and interpretation criteria adopted [29–31]. In 2011, Agius et al. published a prospective study on 11 patients with suspected VGEI (six Dacron prostheses) using microbiology and a follow-up of 6 months as gold standard [29]. A focal or heterogeneous FDG-uptake higher or equal than liver uptake was used as criterion of positivity in PET. ^99m^Tc-WBC scan was interpreted according to EANM recommendations. ^99m^Tc-WBC SPECT/CT and [^18^F]FDG PET/CT provided comparable diagnostic performance (sensitivity: 100% for both and specificity: 100% vs 93%, respectively). Based on these results, the authors suggested to perform [^18^F]FDG PET/CT as first line examination, given its faster execution and wider availability, and to add ^99m^Tc-WBC SPECT/CT, in doubtful cases, in order to definitively rule out or rule in the infection [29].

Conversely, in the largest retrospective study published by Puges et al. in 39 patients, the diagnostic performance and the inter-observer agreement of ^99m^Tc-WBC scintigraphy was statistically higher than [^18^F]FDG PET/CT and CTA [30]. They, therefore, suggested performing ^99m^Tc-WBC scintigraphy after a negative or equivocal CTA and to use [^18^F]FDG PET/CT, as an alternative [30].

More recently, Sollini et al. published a retrospective comparative study in 22 patients with suspected infection of thoracic grafts after Bentall procedure, suggesting the use of ^99m^Tc-WBC SPECT/CT in suspected very early and early infections and the use of [^18^F]FDG PET/CT in all other cases. In line with our view, they suggest confirming or excluding an infection by using the other NM modality as additional examination, in case of unclear findings [31].

Our results confirm that ^99m^Tc-WBC scintigraphy has statistically higher specificity and PPV than [^18^F]FDG PET/CT in detecting an infection.

With ^99m^Tc-WBC SPECT/CT, we observed 2 FN results in patients with proven infection by *Candida tropicalis* and *Pseudomonas aeruginosa*, maybe for the ability of these microorganisms to create biofilm and to escape from host defence. This mechanism is known to reduce the sensitivity of ^99m^Tc-WBC scintigraphy in infective endocarditis [32], and could probably be applied also to VGEI. A low-grade infection could be another possible explanation. No FP results were observed, even in patients studied in early phases after surgery.

[^18^F]FDG PET/CT showed a sensitivity and NPV of 100%. However, our results again confirm that its low specificity is the major drawback of [^18^F]FDG. Despite its biodistribution in non-infected graft is well known [26, 27], a similar consensus on uptake pattern in infected grafts has not been reached yet, especially in case of non-homogeneous uptake, and issue a challenge to improve interpretation of [^18^F]FDG scans.

Different methods for [^18^F]FDG PET/CT interpretation have been proposed [33]. Visual assessment of [^18^F]FDG distribution pattern should always be the first step [23]. Spacek et al. used a subjective evaluation of diffuse or focal [^18^F]FDG uptake by using a three-point scale. They identified focal uptake and irregular graft’s borders as predictive of VGEI [34]. Other authors proposed a five-point visual scale comparing the intensity of [^18^F]FDG uptake with inactive muscles, fat and bladder [35–37]. Overall, these studies reported high sensitivity ranging from 91% [37] to 98% [34] and low-moderate specificity, ranging from 64% [37] to 91% [26].

In their original study, Sah’s scale provided a sensitivity, specificity, accuracy, PPV, and NPV of 100%, 86%, 96%, 100%, and 97%, respectively [28].

By using this scoring system in our study, we also obtained 100% sensitivity and NPV, but we reached lower specificity, accuracy, and PPV (40%, 65%, and 55% respectively), indicating that these criteria are indeed operator dependent. Our new scale provided lower number of FP results compared to Sah’s scale (4 *vs.* 9), thus improving the specificity from 40 to 73% (*p* = 0.049). Overall, this new scale did not emerge to be statistically superior than the previous one, but highlights two important issues that should be further addressed by larger studies: pattern distribution of [^18^F]FDG could be more reliable than its intensity, and some complex cases may benefit from a more detailed scoring system rather than the dichotomy focal *versus* diffuse, since many patients may not fit into any of these categories. Furthermore, this new scale, being more detailed, may reduce operator bias and the number of ^99m^Tc-WBC scintigraphy needed for confirmation of PET findings.

The possible added value of quantification of [^18^F]FDG uptake, over visual assessment, has been largely investigated, being SUVmax the most reported parameter [28, 35, 38–48]. Despite several thresholds for SUVmax have been also proposed [28, 45], a cut-off value able to distinguish, with high accuracy, VGEI from non-infected grafts has not been identified yet. Indeed, also in our study, the ROC curves for semi-quantitative parameters did not show additional value over visual analysis or ^99m^Tc-WBC SPECT/CT, thus further underlying that they have a modest role for imaging infections.

SUVmax values rely on several characteristics including the type of tomographs, incubation time, and reconstruction algorithms, thus making this parameter not reproducible in different centres. Moreover, SUVmax is the maximum activity registered in a single pixel and it is not representative of the whole ROI. Indeed, SUVmax values of infected patients were not different from SUVmax of non-infected patients. Conversely, both SUVmean and T/B ratios were statistically higher in infected patients compared to those without infection. SUVmean is, indeed, the mean of registered counts within a ROI, and it seems more reliable for assessing the activity.

A wide heterogeneity also exists in the methods for calculating T/B ratios (SUVmax graft/SUVmax background, SUVmax graft/SUVmean background, SUVmean graft/SUVmean background) and in the reference tissues to be used as background (liver, caval vein, abdominal or descending aorta), thus resulting in different thresholds [34–37, 41, 45, 49]. Similar to Keidar et al. [26], we calculated T/B ratios by normalizing the mean activity of the graft for the mean activity of blood pool and this showed to be a reliable parameter in discriminating between infected and non-infected patients. Moreover, since no EARL reconstruction was used in the present study, the use of SUVmean appears to be less influenced by the reconstruction algorithms and type of tomograph and, therefore, it could be more reproducible.

But most important, we explored the clinical impact of multimodal imaging in patient’s management.

CTA should be always the first-line imaging modality in suspected VGEI because it is rapidly available, cheap, and provides important anatomic information for the surgeons. However, in several cases, CTA cannot be the only imaging modality. Indeed, in our population, the CTA alone would have determined a wrong management in 27.3% of patients. The addition of NM modalities improved the therapeutic approach, thus further supporting the use of NM in the workup of patients with suspected VGEI after a first radiological assessment.

If we examine again the three different strategies of combination of imaging modalities (Table 7), and interpreting our results in light of patient’s management, the following considerations could be extrapolated:

1. CTA + ^99m^Tc-WBC scintigraphy: given the high PPV of ^99m^Tc-WBC SPECT/CT, a positive scan can reliably diagnose an infection. A PET/CT could be more appropriate in case of positive CTA and negative ^99m^Tc-WBC scintigraphy, due to the risk of fungal or low-grade infections that could be misdiagnosed by ^99m^Tc-WBC scan. With this strategy, 5 patients with negative ^99m^Tc-WBC scan would have needed an additional PET/CT in our population. Nevertheless, two patients were unfortunately considered as infected (both CTA and [^18^F]FDG PET/CT positive) and received an overtreatment;
2. CTA + [^18^F]FDG PET/CT (interpreted according Sah’s scale): given the high sensitivity and NPV of PETC/CT, a negative scan can reliably exclude an infection while a positive scan should require a confirmation with ^99m^Tc-WBC scintigraphy, due to the low specificity of [^18^F]FDG. By using this approach, in our population, 8 patients with negative CTA and positive [^18^F]FDG PET/CT would have required the third examination. The addition of ^99m^Tc-WBC scintigraphy allowed reaching the correct diagnosis in all these 8 patients;
3. CTA + [^18^F]FDG PET/CT (interpreted according to the new scale): with this approach in our population, only 4 patients with negative CTA and positive [^18^F]FDG PET/CT would have required an additional ^99m^Tc-WBC scintigraphy to confirm or rule out the infection. ^99m^Tc-WBC scintigraphy was able to achieve the correct diagnosis in all these 4 patients.

**Fig. 5:**
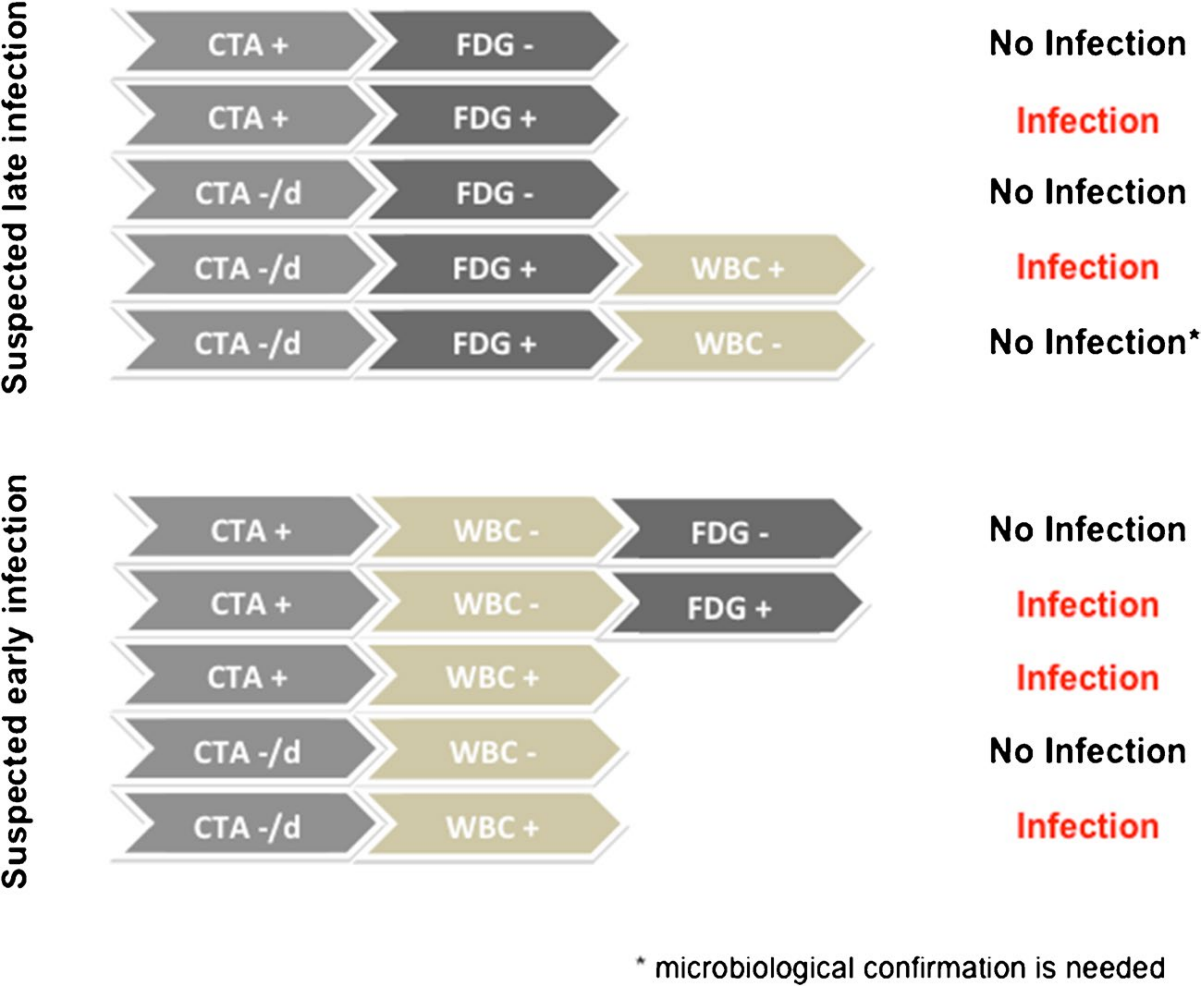
Suggested combination of imaging modalities in patients with suspected VGEI, according to time from surgery. In suspected late infections (more than 4 months from surgery), [^18^F]FDG PET/CT can be successfully used as second imaging modality after performing a CTA. In case of negative or doubtful (d) CTA and positive [^18^F]FDG PET/CT, WBC scintigraphy should be attempted for confirmation. In suspected early infections (within 4 months from surgery), WBC scintigraphy should be the preferred imaging modality after an initial CTA, given its higher specificity in differentiating sterile inflammation from infection. [^18^F]FDG PET/CT should be used in discordant cases

Based on these considerations and in agreement with Agius et al., after an initial CTA, we suggest to start with [^18^F]FDG PET/CT given its high sensitivity, NPV, wider availability, and faster execution (Fig. 5). In our population, the use of a more detailed interpretation criteria resulted in a lower number of discordant cases between CTA and [^18^F]FDG PET/CT and, most important, a lower number of ^99m^Tc-WBC scintigraphies needed for confirmation. Overall, ^99m^Tc-WBC scintigraphy used after [^18^F]FDG PET/CT allowed reaching the correct diagnosis in all the patients given its high ability in differentiating infections from sterile inflammation.

Nevertheless, time from surgery must always be considered. Indeed, with [^18^F]FDG PET/CT, we obtained a FP rate of 40% in five patients studied within four months from surgery.

Therefore, in agreement with the recently published EANM guidelines [16], in suspected early infections, after an initial CTA, we suggest to start with ^99m^Tc-WBC scintigraphy and to perform [^18^F]FDG PET/CT in discordant cases.

Despite relevant results, this study has some limitations: first of all, the small sample size, due to the relative low incidence of VGEI, and the single-centre nature. Nevertheless, we included only patients with suspected infection, which represent, obviously, only a small percentage of patients who undergo to an EVAR procedure. Second, microbiology and histology were not performed in all the patients, but it reflects what usually happens in clinical practice. Indeed, not all patients are eligible for a surgical re-intervention due to the high risk of the procedure and patients’ related conditions. However, we believe that an uneventful clinical, biochemical, and radiological follow-up of more than 2 years is more than appropriate to exclude a VGEI. Third, we could not assess the CTA scans of four patients with only report available, thus, possibly underestimating the impact of this imaging modality.

Although CTA was not an inclusion criterion for our study, it was the first line imaging modality in all the patients. CTA scans were performed in the same referral centre by the majority of the patients and in a different centre by a few of them, as it commonly happens in daily practice. Being the present a prospective comparative study of different modalities, to exclude these patients would have introduced a bias, reduced population’s size and reduced the number of WBCs and [^18^F]FDG PET/CT performed and interpreted with different criteria.

Nevertheless, despite these limitations, the main goals reached by this study were to underline the importance of an integrated and multimodal approach for the correct management of patients with suspected VGEI and to provide potential additional tools for a more specific interpretation of [^18^F]FDG PET/CT.

More prospective studies with larger sample size are needed to eventually confirm our results and to define more robust interpretation criteria for [^18^F]FDG PET/CT.

## Conclusions

NM imaging modalities should be always included in the workup of patients with suspected VGEI after a CTA.

Based on our results, we recommend performing [^18^F]FDG PET/CT as second modality, due to its high sensitivity and NPV, unless the time from surgery is less than 4 months. In this case, it is more convenient to perform a ^99m^Tc-WBC SPECT/CT after CTA.

Positive [^18^F]FDG PET/CT should be interpreted with caution, due to the high number of FP cases and should be confirmed by ^99m^Tc-WBC SPECT/CT.

The new visual scale, that gives more emphasis to the pattern of [^18^F]FDG distribution rather than the intensity of uptake, increases the specificity.

## Data Availability

Data are available on request.
